# Triple Diagnosis of Attention-Deficit/Hyperactivity Disorder with Co-existing Bipolar and Alcohol Use Disorders: Clinical Aspects and Pharmacological Treatments

**DOI:** 10.2174/1570159X20666220830154002

**Published:** 2023-05-18

**Authors:** Francesco Weiss, Simone Tidona, Marco Carli, Giulio Perugi, Marco Scarselli

**Affiliations:** 1 Psychiatry Unit 2, Department of Clinical and Experimental Medicine, Faculty of Medicine and Surgery, University of Pisa, Pisa, Italy;; 2 Department of Translational Research and New Technologies in Medicine and Surgery, University of Pisa, Pisa 56126, Italy

**Keywords:** ADHD, alcohol use disorder, bipolar disorder, triple diagnosis, neurodevelopment, lithium, valproic acid

## Abstract

Attention-Deficit/Hyperactivity Disorder (ADHD), Bipolar Disorder (BD) and Alcohol Use Disorder (AUD) are common medical conditions often coexisting and exerting mutual influence on disease course and pharmacological treatment response. Each disorder, when considered separately, relies on different therapeutic approaches, making it crucial to detect the plausible association between them. Treating solely the emerging condition (*e.g*., alcoholism) and disregarding the patient’s whole psychopathological ground often leads to treatment failure and relapse. Clinical experience and scientific evidence rather show that tailoring treatments for these three conditions considering their co-occurrence as a sole complex disorder yields more fulfilling and durable clinical outcomes. In light of the above considerations, the purpose of the present review is to critically discuss the pharmacological strategies in the personalized treatment of complex conditions defined by ADHD-bipolarity-alcoholism coexistence.

## 1 INTRODUCTION

Bipolar disorder (BD) is a severe psychiatric and medical condition, associated with generalized psychosocial impairment, increased risk of physical illness and suicide, substance abuse and a reduced life expectancy. BD is characterized by lifetime cycles of affective episodes, roughly described as depressive, expansive or mixed, alternating with asymptomatic intervals, the so-called euthymic periods [1]. Although epidemiological data vary greatly across studies, it is estimated that the overall lifetime prevalence of bipolarity (including bipolar spectrum) reaches up to 4.8%. A large number of patients with BD, if thoroughly investigated, display features of neurodevelopmental disorders, particularly Attention-Deficit/Hyperactivity Disorder (ADHD) symptoms. ADHD is a heterogeneous neurodevelopmental disorder characterized by a variable combination of inattentive and hyperactivity symptoms, along with dysexecutive deficits and emotional dysregulation [2]. Its worldwide estimated prevalence ranges from 3% to 5% in children and 1.4% to 3.6% in adults [3]. This condition shows a discontinued and dynamic course through patients’ life, being subject to relatively different and, to a certain extent, predictable behavioral trajectories, which largely modify the clinical picture from its onset to adulthood and make its diagnosis difficult [4, 5]. ADHD is burdened with a heavy psychiatric comorbidity, being strongly associated with both mood disorders, especially BD, and substance use disorder (SUD). It is estimated that the risk to develop SUD is increased by almost twelve times in people with a diagnosis of ADHD, while the risk to develop BD being increased by up to twenty-four times [6]. In addition, both BD and ADHD have been shown to frequently co-occur with cluster B personality disorders (particularly antisocial personality and borderline personality disorders), which are in turn independently associated with SUDs, particularly alcohol use disorder (AUD), further complicating the phenomenological presentation and management of these patients [7-9]. Subjects with ADHD tend to develop SUD at younger ages, and the risk seems to be increased by some specific factors, such as novelty seeking traits, scholastic failures, social difficulties and isolation, conduct disorder and BD comorbidity [10-12]. Although earlier studies showed a stronger developmental association between childhood ADHD and SUD or conduct/antisocial personality disorders than with affective disorders [13], more recent accounts have highlighted specific risk factors for its “progression” to BD [14]. These include comorbidity with autism spectrum disorders, disruptive behavior disorders (namely, opponent defiant disorder and conduct disorder), alcohol use disorder, cluster A and B personality disorders and a family history of BD. Conversely, male sex and cluster C personality seem to be protective. As a consequence, many ADHD adult patients present very complex and intertwined maladaptive behavioral problems, complicating the clinical features of mood disorder with earlier onset and longer duration of episodes, and higher rates of suicidality [15]. In particular, a still controversial issue is the influence of ADHD on the pharmacological treatment of patients diagnosed with BD and SUD. AUD still represents nowadays one of the most common addiction disorders, although it goes often underdiagnosed and undertreated, with limited evidence-based pharmacotherapeutic approaches [16]. In comparison to other SUDs, AUD exhibits unique socio-psychological profiles: alcoholic beverages are easily and legally purchasable in almost every developed country, with ethanol being commonly consumed in social contests tied to ancient and culturally determined traditions. Inappropriate alcohol usage, such as binge-drinking and drunkorexia [17], and alcohol-related dangerous behaviors, such as drunk driving, are ordinarily observed in our everyday life, especially in the youth. Expectedly, AUD is strongly associated with other neuropsychiatric conditions such as non-alcohol SUD, mood disorders, and ADHD [11, 18]. With these premises, the present review critically discusses the pharmacological strategies and clinical aspects of the complex condition defined by ADHD-bipolarity-alcoholism triple diagnosis.

## 2 GENETICS, GENDER AND NEUROPSYCHO-BIOLOGY OF COEXISTING ADHD-AUD-BD

All these three conditions exhibit high rates of heritability, ranging from 60% up to 90% in BD [19, 20], about 70-80% for ADHD [21] and 50-60% for AUD [22]. Although BD has traditionally been reputed as a sex/gender-unrelated condition, more recent sources seem to point to a role of this variable in the etiology and phenomenology of BD. In fact, women seem to be at greater risk of developing BD (especially BD type II) and of complicated phenotypes, *e.g*. in terms of rapid cycling, mixed states, peri/post-partum episodes and suicidality [23]. ADHD is more frequently diagnosed in boys than in girls, but such difference is likely ascribable to a selection bias: girls are less likely to be referred to a specialist since female ADHD phenotypes are associated with fewer externalizing symptoms of the hyperactivity/impulsivity group, which are often perceived as the most problematic. Accordingly, sex differences seem to subside in adult populations, arguably due to the greater persistence in age transition of inattentive/dysexecutive deficits over hyperactive/impulsive behaviors [24]. Historically, there has always been a greater prevalence of SUD in men than in women. However, gender gaps are progressively narrowing throughout the last decades, and AUD is no exception [25]. Considering that the available evidence points out a significant genetic overlap between these disorders [11, 26, 27], their co-existence is subtended by remarkable inborn causation, with a variable contribution of gender-related pathoplastic influences on disease phenotypes. Interestingly, although the prevalence of AUD seems to be greater in men with BD than in women with BD, the association between BD and AUD seems to be significantly stronger in women [28]. A possible pathomechanistic pathway which could entangle all of these three conditions in a single rational design is the so-called “addiction cycle” theoretical framework, proposed and implemented by Koob and coworkers [29]. According to the authors, SUDs are chronic-progressive relapsing brain diseases resulting from a vicious loop of three pathogenic moments: executive dysfunction, negative affection and disordered incentive salience (*i.e*., craving and amotivational syndrome) [30]. ADHD is by definition associated with variable degrees of executive dysfunction, emotional dysregulation and reward system abnormalities, and thus could intercept and facilitate all of the three moments of this cycle [31, 32]. As to BD, this syndrome is frequently associated with both inter-episodic and intra-episodic behavioral features that could promote addiction cycle initiation and progression. During free intervals, subjects with BD tend to exhibit strong temperamental traits [33, 34], especially cyclothymic traits [35], which could worsen and be worsened by the negative emotionality state that defines drug dependence (hyperkatifeia) [36]. During expansive phases, patients could be prone to impulsive or reckless choices and actions [37]. During dysphoric mixed phases, increased impulsivity is accompanied by elevated anxiety [38]. Co-existing ADHD and BD could thus entail the superimposition of state variables (*e.g*. state impulsivity, state anxiety *etc*.), which are definitory of mood cycling, over trait variables attached to neurodevelopmental divergencies, and affective temperamental dimensions. Finally, a “reward deficiency syndrome,” based either on genetic (especially D2 receptor) or acquired mechanisms [39], could link AUD and ADHD together and intercept the salience disorder moment of the cycle.

## 3 A BRIEF EXCURSUS ON THE PHARMACOLOGICAL TREATMENTS OF AUD

Harmful alcohol consumption is a major cause of mortality and morbidity worldwide, with 5.1% of the global disease burden and 5.3% of all deaths attributable to alcohol misuse, according to WHO 2018 report [40]. AUD is a very heterogeneous and multicausal condition, with ethanol-addicted patients being very different from each other [41]. Thus, the management of this condition remains defying and often unsatisfactory, especially when not assisted by a careful psychopathological examination and subsequent treatment of the most frequently associated disorders, such as mood and neurodevelopmental disorders. In addition, the currently available pharmacological tools are generally more efficacious in reducing the risk of relapse in already detoxified patients, rather than inducing or favoring abstinence, often requiring a previous acute phase of withdrawal management with benzodiazepines (with diazepam as the gold standard) and, if needed, adjunct therapy with sodium oxybate (in Italy and Austria), alpha2-agonists (clonidine) and beta-blockers (atenolol) [42]. Drugs used in Europe for long-term treatment of AUD include disulfiram, acamprosate, naltrexone (oral and extended-release), nalmefene, baclofen (in France) [16]. In the U.S. only the first three drugs are FDA-approved for the treatment of alcoholism. Other promising repurposed pharmaceuticals, sometimes used “off-label”, in the treatment of AUD include the anticonvulsants topiramate and gabapentin, the 5-HT3 antagonist ondansetron, the nicotinic agonist varenicline and the alpha-1 antagonists prazosin and doxazosin [16]. Pregabalin may be a promising therapeutic agent for the treatment of alcohol abuse, in both the withdrawal phase and relapse prevention [43], shown to be safe and well tolerated [44]. Moreover, results from a randomized, double-blind comparison trial with naltrexone, place these two drugs within the same range of efficacy [45]. Interestingly, topiramate has demonstrated clinical efficacy in reducing alcohol consumption in still drinking patients, rather than solely preventing relapse after complete detoxification [46]. Ketamine, a psychodysleptic dissociative substance that has long been experimented with on a large scale for the treatment of resistant major depression, has been reported to show some promising results in the treatment of AUD [47, 48], but no study was carried out in patients with psychiatric comorbidity [49].

## 4 ADHD AND AUD: A COMMON, YET NEGLECTED, ASSOCIATION

Albeit most reviews on ADHD comorbidities deal with the bidirectional relationship between ADHD and SUD without a specific reference to AUD, epidemiological data suggest that AUD is commonly associated with ADHD, with an estimated lifetime prevalence of 43% in this population [50], and it is likely responsible for more complicated disease trajectories and unsatisfactory responses to treatments [11]. Conversely, ADHD seems to co-occur in up to 20% of all patients with AUD [51]. Even though the ADHD-AUD co-occurrence might be less specific than those with other SUDs, it represents a clinically impactful association on account of the widespread use of ethanol and of the significant morbidity and mortality therewith correlated [11, 40]. Additionally, as observed for SUDs in general, ADHD and AUD seem to be genetically linked and exhibit familial coaggregation. In particular, higher polygenic risk scores for ADHD are associated with an augmented risk of AUD [52]. The psychopathological link between ADHD and AUD can be viewed as bidirectional, although neurodevelopmental disorders must be logically conceived as a starting point in the neuropsychiatric trajectory. Multiple psychogenic pathways that might lead from ADHD to AUD have been hypothesized. For example, people with ADHD often display an increased vulnerability to subjectively stressful and traumatic events and are at greater risk of post-traumatic stress symptoms and alcohol misuse [53, 54]. Most likely, emotional dysregulation, difficulties in managing reward directed behaviors and impulsivity might represent important psychopathological bridge between ADHD, stress sensitivity and AUD [55]. Impulsivity is one of the core features of ADHD syndrome, and it has been independently associated with SUDs and alcohol misuse [56, 57]. Impulsivity might be conceived either as a trait variable, intrinsically linked to neurodevelopment and education, or as a state variable, which can be influenced by functional phases (such as mood episodes) or toxic drug-induced conditions. State impulsivity overlies trait impulsivity, modifying and possibly exacerbating impulsive behaviors in predisposed individuals [56]. For example, subjects with ADHD seem to be more sensitive to the disinhibiting/pro-impulsive effects of ethanol than neurotypical individuals [58]. In addition, heavy alcohol consumption, especially during adolescence, has been shown to entail a further and seemingly persistent neurodevelopmental impairment in prepotent response inhibition, worsening trait impulsivity [59]. Thus, the relationship between impulsivity and alcohol abuse seems to be mutual and bidirectional. Finally, both ADHD and AUD seem to be associated with a hypodopaminergic state and a reduced reward sensitivity [11]. Detoxified individuals with AUD exhibit a much lower striatal release of dopamine following the administration of methylphenidate, suggesting a persistently reduced dopaminergic transmission in this population [60]. Another alteration of reward processing fairly typical of subjects with ADHD is delay discounting, that is, the tendency to undervalue a delayed gratification and choose the immediate fulfilling of smaller, maladaptive or unproductive rewards [61]. Temporal discounting could be put in relation with an increased sensitivity to the immediate reinforcing effects of alcohol use, that is perceived as pleasure-inducing and/or discomfort-relieving [30].

## 5 AUD IN BD CONSIDERING NEURO-DEVELOP-MENT: PHENOMENOLOGY OF CO-OCCURRENCE

BD is frequently associated with both alcohol and non-alcohol SUD, but AUD is for sure one of the most common in the western world. It is estimated that almost one quarter of patients suffering from BD have coexisting AUD, with an estimated lifetime comorbidity prevalence of 40-70% [62-64]. In turn, subjects with AUD show a 4.1 times increased risk of having BD [65]. AUD in patients with BD is associated with a more complicated clinical picture, including greater affective instability, impulsivity, suicidality, hospitalization rate, cognitive decline, a higher incidence of mixed-dysphoric states, a worse disease course with a tendency to rapid cycling, and a poorer response to mood stabilizing treatments [66-69]. According to some sources, AUD might even be the first cause of treatment nonadherence among patients with BD [70, 71]. Considering that treatment non-adherence in patients with BD shows a median prevalence of 40% (range 20-66%) [71], and that ADHD-related dysexecutive cognitive deficits might further impair patients’ capability of adhering to a treatment program [72], it can be readily understood that recognizing triple co-occurrence is a crucial factor to reduce the risk of non-adherence to pharmacological treatments. Suicidality is another critical issue when it comes to co-occurrent disorders, since both ADHD and AUD are strong independent risk factors for suicidal behaviors, arguably synergistically increasing the already elevated risk for self-harming posed by mood states in BD [73-76]. In fact, BD is notoriously the psychiatric condition bearing the highest incidence of suicidality, estimated to be up to 20-30 times greater than the general population, and about 20% of BD patients die because of completed suicide [77, 78]. Even though statistical data about suicidality in triple comorbidity are lacking, it is presumable that patients with complex co-occurrent disorders should be clinically considered as the population with a very high risk of suicidal behaviors.

## 6 TO KILL TWO BIRDS WITH ONE STONE: A CONCEPTUAL PROPOSAL OF MANAGEMENT GUIDELINES FOR TRIPLE DIAGNOSIS

**Fig. (1):**
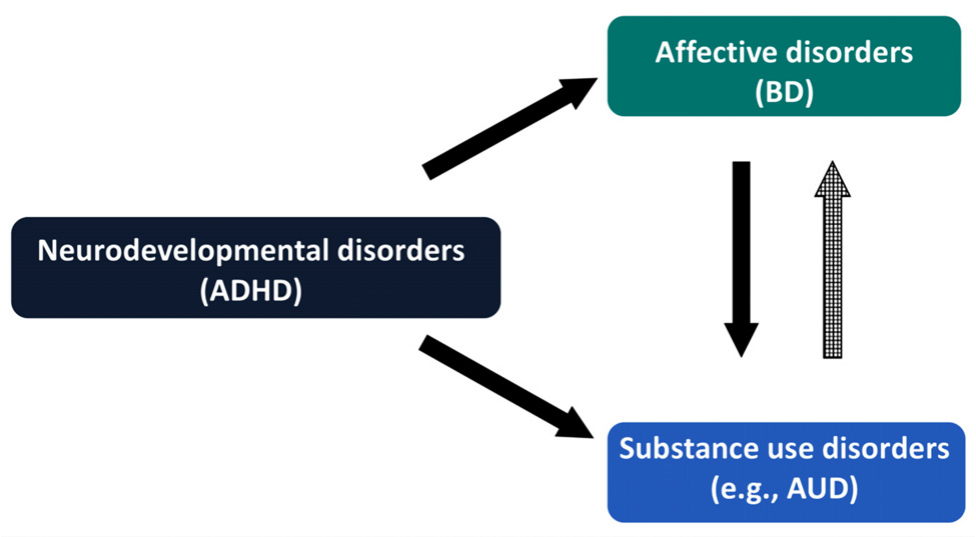
Triple diagnosis of AUD with co-existing BD and ADHD, characterized by a plausible trajectory starting from ADHD and evolving to BD and AUD.

As mentioned beforehand, BD is often complicated with SUD, including AUD, and is strongly associated with neurodevelopmental disorders, such as ADHD (Fig. **1**).

**Fig. (2):**
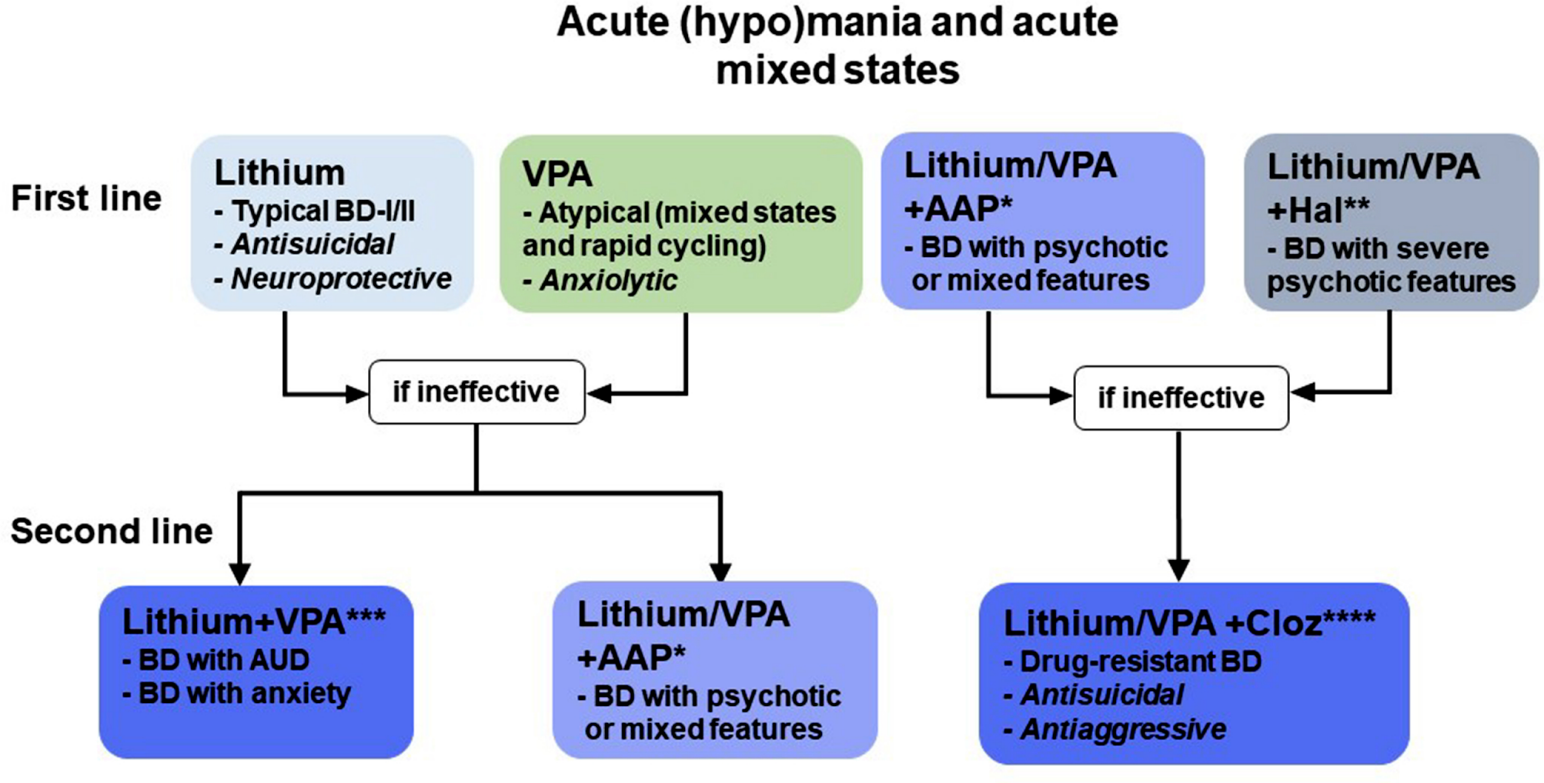
Pharmacological treatments for AUD, AUD/BD and AUD/BD/ADHD.

SUD/AUD may obscure the diagnosis of BD in many healthcare contexts, leaving patients in incomplete management and treatment programs. In addition, BD with SUD tends to show high rates of relapse, rapid/continuous cycling and reduced response to mood-stabilizers [79]. On the other hand, mood disorders treatment, especially with lithium or valproic acid, might reduce the probability of SUD/AUD relapse [80]. Unfortunately, there is a dearth of literature concerning the treatment strategies and predictors of response in patients with complex disorders. In fact, most randomized controlled studies concerning the treatment of BD exclude patients with comorbid SUD, denying relevant evidence for real-world clinical practice [81]. Since depressive episodes with AUD or other SUD are strongly suggestive of bipolarity, another theoretical problem is missed BD diagnosis in patients with AUD, instead labeled with unipolar depression, and consequently treated with antidepressants in the absence of an adequate mood stabilizing strategy [82-85]. Siding with other authors, the fundamental idea is to question the single-disease model of psychiatric treatment and embrace an integrated care approach for complex disorders [86, 87]. Since it is reasonable to conceive a disease trajectory starting from ADHD and evolving into affective disorders (in particular BD), both predisposing to SUD and AUD, early detection and treatment of ADHD features might prove efficacious in preventing further progression into complex “triple” disorders. As a matter of fact, full dose psychostimulant treatment during childhood has been shown to reduce SUD risk, including AUD and Nicotine Use Disorder [88-92]. Anyway, in medical practice, clinicians often deal with already complex conditions in adult patients, which often require the short-term management of acute phases (*i.e*., intoxication or withdrawal, mania, mixed states, depression, suicide attempts) and then should progress to a long-term treatment planning aimed at preventing mood/substance use relapses and extending euthymic intervals. With these premises, we propose a hierarchical model of long-term treatment in adult complex patients, wherein mood relapse prevention is the first therapeutic target, often conditioning other clinical outcomes and generally representing a necessary prerequisite for sustained remission (Fig. **2**). In support of this approach, there is initial evidence that appropriate mood stabilization with commonly used mood-regulating agents, such as lithium and valproic acid, reduces AUD relapse rates on its own in monotherapy and even more in association [93-95]. For lithium, few studies have reported the efficacy of lithium versus placebo in BD and co-occurring SUD [80]. A 24-week RCT on BD/AUD patients taking lithium and then treated with valproic acid add-on registered a significant reduction in the number of heavy drinking days and fewer drinks per heavy drinking day in treated patients [93]. Lithium might have an anti-abuse/anti-impulsive effect in complex patients with SUD, independently of the stabilizing effect on comorbid psychiatric diagnoses, enhancing self-constructive behaviors and improving psychosocial outcomes [96-99]. Even though lamotrigine seems promising as a combined mood-stabilizer/abuse-preventing agent, the available evidence is even weaker [100-102]. There are no studies of carbamazepine and oxcarbazepine in the treatment of BD complicated with AUD, although there are data supporting a possible role of carbamazepine as an anti-impulsive and anti-abuse agent in patients with cocaine use disorder [103]. However, there is also evidence that oxcarbazepine, especially at full doses, might show some benefit in preventing AUD relapses, even when compared to specific anti-abuse agents, such as naltrexone and acamprosate [104-107]. A 2016 study has reported that topiramate, despite the promising results mentioned above in the treatment of AUD, seems to be ineffective in BD patients with AUD [108]. However, given the small sample size, this observation still needs to be confirmed. Quetiapine, although proven useful in the treatment and prevention of both manic and depressive episodes [109], has consistently yielded no benefit over placebo in preventing AUD relapses in patients with BD [110-112]. Despite the burdensome probability of therapeutic non-adherence in these complex patients, there are no specific indications for the employment of long acting injectable antipsychotics in AUD/SUD relapse prevention [113].

Add-on studies of naltrexone in patients with comorbid BD and AUD have shown promising results in reducing drinking outcomes as well as affective symptoms burden [114, 115]. Conversely, add-on acamprosate has not been associated with any clinical advantage over placebo [116]. In general, ADHD treatment could concomitantly reduce substance abuse risk, but the issue is still controversial [87, 117]. There is only one study suggesting that atomoxetine could have a modest effect in reducing heavy drinking in adult patients with comorbid ADHD and AUD, but data are still limited, and further investigation is required [118]. The role of psychostimulants in the treatment of SUD in adult ADHD appears more debatable and seemingly limited to the prevention of relapse [87]. However, some studies reported that medications for ADHD improve the clinical course of both ADHD and SUD [119, 120].

ADHD pharmacotherapy was associated with improved short- and longer-term retention in patients in treatment for SUD [121], and in one study, no difference was found between ADHD and ADHD/SUD patients on this matter [122]. The extent to which ADHD medications, namely methylphenidate and atomoxetine, increase the risk of mood switches is still a matter of debate [123]. The risk of switches is particularly high when these drugs are not co-administered with mood stabilizers [124]. However, the co-administration of mood stabilizers is partially protective. In this respect, stimulants seem to be less associated with mood switches than atomoxetine [125]. A large Taiwanese controlled register study on 145,000 children with a recent diagnosis of ADHD reported no association between ADHD treatment initiation and BD onset, with an even reduced risk in patients treated with methylphenidate [126]. In naturalistic studies, methylphenidate showed efficacy in affective symptoms in patients with BD [127, 128]. In another recent study with 2307 adults with BD treated with methylphenidate and a concomitant mood stabilizer, the risk of mania was strongly reduced at 3 and 6 months compared to the single treatment [129]. According to the recent Canadian guidelines, bupropion is another valuable option to treat BD with co-occurring ADHD, especially considering its probable efficacy on inattentive and addictive symptoms, along with a low risk of inducing manic switches [130].

## CONCLUSION

In conclusion, the present work tried to pinpoint the deleterious clinical consequences of disregarding psychiatric co-occurrences and their hierarchical relationships. There is evidence that ADHD, BD and AUD are tightly connected to each other, both in psychopathological and neurobiological terms. Treating state disturbances linked to affective cycling and trait features pertaining to temperamental and neurodevelopmental dimensions is crucial for therapeutic success and remission endurance. Accordingly, there is preliminary evidence that classical mood-stabilizing agents (*i.e*., lithium salts and valproic acid) are to be viewed as the cornerstone of the treatment of complex conditions defined by a layered neurodevelopment-bipolarity-addiction triple diagnosis.

## LIST OF ABBREVIATIONS

AUD: Alcohol Use Disorder
ADHD: Attention-Deficit/Hyperactivity Disorder
BD: Bipolar Disorder
SUD: Substance Use Disorder

## References

[1] Fountoulakis K.N. (2015). Clinical description BT-bipolar disorder: An evidence-based guide to manic depression..

[2] Barkley R.A. (2015). Attention-deficit hyperactivity disorder: A handbook for diagnosis and treatment..

[3] Kooij J.J.S., Bijlenga D., Salerno L., Jaeschke R., Bitter I., Balázs J., Thome J., Dom G., Kasper S., Nunes Filipe C., Stes S., Mohr P., Leppämäki S., Casas M., Bobes J., Mccarthy J.M., Richarte V., Kjems Philipsen A., Pehlivanidis A., Niemela A., Styr B., Semerci B., Bolea-Alamanac B., Edvinsson D., Baeyens D., Wynchank D., Sobanski E., Philipsen A., McNicholas F., Caci H., Mihailescu I., Manor I., Dobrescu I., Saito T., Krause J., Fayyad J., Ramos-Quiroga J.A., Foeken K., Rad F., Adamou M., Ohlmeier M., Fitzgerald M., Gill M., Lensing M., Motavalli Mukaddes N., Brudkiewicz P., Gustafsson P., Tani P., Oswald P., Carpentier P.J., De Rossi P., Delorme R., Markovska Simoska S., Pallanti S., Young S., Bejerot S., Lehtonen T., Kustow J., Müller-Sedgwick U., Hirvikoski T., Pironti V., Ginsberg Y., Félegyházy Z., Garcia-Portilla M.P., Asherson P. (2019). Updated European Consensus Statement on diagnosis and treatment of adult ADHD.. Eur. Psychiatry.

[4] Vos M., Rommelse N.N.J., Franke B., Oosterlaan J., Heslenfeld D.J., Hoekstra P.J. (2021). Characterizing the heterogeneous course of in attention and hyperactivity-impulsivity from childhood to young adulthood.. Eur. Child Adolesc. Psychiatry.

[5] Sibley M.H., Arnold L.E., Swanson J.M., Hechtman L.T., Kennedy T.M., Owens E., Molina B.S.G., Jensen P.S., Hinshaw S.P., Roy A., Chronis-Tuscano A., Newcorn J.H., Rohde L.A. (2022). Variable patterns of remission from ADHD in the multimodal treatment study of ADHD.. Am. J. Psychiatry.

[6] Chen Q., Hartman C.A., Haavik J., Harro J., Klungsøyr K., Hegvik T.A., Wanders R., Ottosen C., Dalsgaard S., Faraone S.V., Larsson H. (2018). Common psychiatric and metabolic comorbidity of adult attention-deficit/hyperactivity disorder: A population-based cross-sectional study.. PLoS One.

[7] Helle A., Watts A.L., Trull T.J., Sher K.J. (2019). Alcohol use disorder and antisocial and borderline personality disorders.. Alcohol Res..

[8] Latalova K., Prasko J., Kamaradova D., Sedlackova J., Ociskova M. (2013). Comorbidity bipolar disorder and personality disorders.. Neuroendocrinol. Lett..

[9] Matthies S., Philipsen A. (2016). Comorbidity of personality disorders and adult attention deficit hyperactivity disorder (ADHD)—review of recent findings.. Curr. Psychiatry Rep..

[10] Magon R., Müller U. (2012). ADHD with comorbid substance use disorder: Review of treatment.. Adv. Psychiatr. Treat..

[11] Luderer M., Ramos Quiroga J.A., Faraone S.V., Zhang-James Y., Reif A. (2021). Alcohol use disorders and ADHD.. Neurosci. Biobehav. Rev..

[12] Molina B.S.G., Pelham W.E. (2014). Attention-deficit/hyperactivity disorder and risk of substance use disorder: developmental considerations, potential pathways, and opportunities for research.. Annu. Rev. Clin. Psychol..

[13] Klein R.G., Mannuzza S. (1991). Long-term outcome of hyperactive children: a review.. J. Am. Acad. Child Adolesc. Psychiatry.

[14] Chu C.S., Tsai S.J., Hsu J.W., Huang K.L., Cheng C.M., Su T.P., Chen T.J., Bai Y.M., Liang C.S., Chen M.H. (2021). Diagnostic progression to bipolar disorder in 17,285 adolescents and young adults with attention deficit hyperactivity disorder: A longitudinal follow-up study.. J. Affect. Disord..

[15] Regnart J., Truter I., Meyer A. (2017). Critical exploration of co-occurring Attention-Deficit/Hyperactivity Disorder, mood disorder and Substance Use Disorder.. Expert Rev. Pharmacoecon. Outcomes Res..

[16] Burnette E.M., Nieto S.J., Grodin E.N., Meredith L.R., Hurley B., Miotto K., Gillis A.J., Ray L.A. (2022). Novel agents for the pharmacological treatment of alcohol use disorder.. Drugs.

[17] Lupi M., Martinotti G., Di Giannantonio M. (2017). Drunkorexia: an emerging trend in young adults.. Eat. Weight Disord..

[18] Grant B.F., Goldstein R.B., Saha T.D., Chou S.P., Jung J., Zhang H., Pickering R.P., Ruan W.J., Smith S.M., Huang B., Hasin D.S. (2015). Epidemiology of DSM-5 Alcohol Use Disorder.. JAMA Psychiatry.

[19] Smoller J.W., Finn C.T. (2003). Family, twin, and adoption studies of bipolar disorder.. Am. J. Med. Genet. C. Semin. Med. Genet..

[20] Barnett J.H., Smoller J.W. (2009). The genetics of bipolar disorder.. Neuroscience.

[21] Faraone S.V., Larsson H. (2019). Genetics of attention deficit hyperactivity disorder.. Mol. Psychiatry.

[22] Reilly M.T., Noronha A., Goldman D., Koob G.F. (2017). Genetic studies of alcohol dependence in the context of the addiction cycle.. Neuropharmacology.

[23] Dell’Osso B., Cafaro R., Ketter T.A. (2021). Has bipolar disorder become a predominantly female gender related condition? Analysis of recently published large sample studies.. Int. J. Bipolar Disord..

[24] Mowlem F.D., Rosenqvist M.A., Martin J., Lichtenstein P., Asherson P., Larsson H. (2019). Sex differences in predicting ADHD clinical diagnosis and pharmacological treatment.. Eur. Child Adolesc. Psychiatry.

[25] White A. (2020). Gender differences in the epidemiology of alcohol use and related harms in the United States.. Alcohol Res..

[26] O’Connell K.S., Shadrin A., Bahrami S., Smeland O.B., Bettella F., Frei O., Krull F., Askeland R.B., Walters G.B., Davíðsdóttir K., Haraldsdóttir G.S., Guðmundsson Ó.Ó., Stefánsson H., Fan C.C., Steen N.E., Reichborn-Kjennerud T., Dale A.M., Stefánsson K., Djurovic S., Andreassen O.A. (2021). Identification of genetic overlap and novel risk loci for attention-deficit/] hyperactivity disorder and bipolar disorder.. Mol. Psychiatry.

[27] Wiström E.D., O’Connell K.S., Karadag N., Bahrami S., Hindley G.F.L., Lin A., Cheng W., Steen N.E., Shadrin A., Frei O., Djurovic S., Dale A.M., Andreassen O.A., Smeland O.B. (2022). Genome‐wide analysis reveals genetic overlap between alcohol use behaviours, schizophrenia and bipolar disorder and identifies novel shared risk loci.. Addiction.

[28] Frye M.A., Altshuler L.L., McElroy S.L., Suppes T., Keck P.E., Denicoff K., Nolen W.A., Kupka R., Leverich G.S., Pollio C., Grunze H., Walden J., Post R.M. (2003). Gender differences in prevalence, risk, and clinical correlates of alcoholism comorbidity in bipolar disorder.. Am. J. Psychiatry.

[29] Koob G.F., Moal M.L. (1997). Drug abuse: hedonic homeostatic dysregulation.. Science.

[30] Koob G.F., Volkow N.D. (2016). Neurobiology of addiction: A neurocircuitry analysis.. Lancet Psychiatry.

[31] Volkow N.D., Wang G-J., Newcorn J.H., Kollins S.H., Wigal T.L., Telang F., Fowler J.S., Goldstein R.Z., Klein N., Logan J., Wong C., Swanson J.M. (2011). Motivation deficit in ADHD is associated with dysfunction of the dopamine reward pathway.. Mol. Psychiatry.

[32] Petrovic P., Castellanos F.X. (2016). Top-down dysregulation—from ADHD to emotional instability.. Front Behav Neurosci..

[33] Cassano G.B., Akiskal H.S., Perugi G., Musetti L., Savino M. (1992). The importance of measures of affective temperaments in genetic studies of mood disorders.. J. Psychiatr. Res..

[34] Perugi G., Toni C., Maremmani I., Tusini G., Ramacciotti S., Madia A., Fornaro M., Akiskal H.S. (2012). The influence of affective temperaments and psychopathological traits on the definition of bipolar disorder subtypes: A study on Bipolar I Italian National sample.. J. Affect. Disord..

[35] Perugi G., Hantouche E., Vannucchi G. (2017). Diagnosis and treatment of cyclothymia: The “Primacy” of temperament.. Curr. Neuropharmacol..

[36] Koob G.F. (2021). Drug Addiction: Hyperkatifeia/negative reinforcement as a framework for medications development.. Pharmacol. Rev..

[37] Messer T., Lammers G., Müller-Siecheneder F., Schmidt R.F., Latifi S. (2017). Substance abuse in patients with bipolar disorder: A systematic review and meta-analysis.. Psychiatry Res..

[38] Di Nicola M., Pepe M., Modica M., Lanzotti P., Panaccione I., Moccia L., Janiri L. (2020). Mixed states in patients with substance and behavioral addictions.. Psychiatr. Clin. North Am..

[39] Blum K., Gondré-Lewis M.C., Baron D., Thanos P.K., Braverman E.R., Neary J., Elman I., Badgaiyan R.D. (2018). Introducing precision addiction management of reward deficiency syndrome, the construct that underpins all addictive behaviors.. Front. Psychiatry.

[40] Poznyak V., Rekve D., World Health Organization. (2018). Global status report on alcohol and health 2018.

[41] Litten R.Z., Ryan M.L., Falk D.E., Reilly M., Fertig J.B., Koob G.F. (2015). Heterogeneity of alcohol use disorder: Understanding mechanisms to advance personalized treatment.. Alcohol. Clin. Exp. Res..

[42] Witkiewitz K., Litten R.Z., Leggio L. (2019). Advances in the science and treatment of alcohol use disorder.. Sci. Adv..

[43] Martinotti G., Lupi M., Sarchione F., Santacroce R., Salone A., Berardis D., Serroni N., Cavuto M., Signorelli M., Aguglia E., Valchera A., Iasevoli F., Giannantonio M. (2013). The potential of pregabalin in neurology, psychiatry and addiction: A qualitative overview.. Curr. Pharm. Des..

[44] Mariani J.J., Pavlicova M., Choi C.J., Brooks D.J., Mahony A.L., Kosoff Z., Naqvi N., Brezing C., Luo S.X., Levin F.R. (2021). An open-label pilot study of pregabalin pharmacotherapy for alcohol use disorder.. Am. J. Drug Alcohol Abuse.

[45] Martinotti G., Di Nicola M., Tedeschi D., Andreoli S., Reina D., Pomponi M., Mazza M., Romanelli R., Moroni N., De Filippis R., Di Giannantonio M., Pozzi G., Bria P., Janiri L. (2010). Pregabalin versus naltrexone in alcohol dependence: A randomised, double-blind, comparison trial.. J. Psychopharmacol..

[46] Kenna G., Lomastro T., Schiesl A., Leggio L., Swift R. (2009). Review of topiramate: An antiepileptic for the treatment of alcohol dependence.. Curr. Drug Abuse Rev..

[47] Das R.K., Gale G., Walsh K., Hennessy V.E., Iskandar G., Mordecai L.A., Brandner B., Kindt M., Curran H.V., Kamboj S.K. (2019). Ketamine can reduce harmful drinking by pharmacologically rewriting drinking memories.. Nat. Commun..

[48] Dakwar E., Levin F., Hart C.L., Basaraba C., Choi J., Pavlicova M., Nunes E.V. (2020). A single ketamine infusion combined with motivational enhancement therapy for alcohol use disorder: A randomized midazolam-controlled pilot trial.. Am. J. Psychiatry.

[49] Martinotti G., Chiappini S., Pettorruso M., Mosca A., Miuli A., Di Carlo F., D’Andrea G., Collevecchio R., Di Muzio I., Sensi S.L., Di Giannantonio M. (2021). Therapeutic potentials of ketamine and esketamine in Obsessive–Compulsive Disorder (OCD), Substance Use Disorders (SUD) and Eating Disorders (ED): A review of the current literature.. Brain Sci..

[50] Tuithof M., ten Have M., van den Brink W., Vollebergh W., de Graaf R. (2012). The role of conduct disorder in the association between ADHD and alcohol use (disorder). Results from the Netherlands Mental Health Survey and incidence study-2.. Drug Alcohol Depend..

[51] Luderer M., Sick C., Kaplan-Wickel N., Reinhard I., Richter A., Kiefer F., Weber T. (2020). Prevalence estimates of ADHD in a sample of inpatients with alcohol dependence.. J. Atten. Disord..

[52] Wimberley T., Agerbo E., Horsdal H.T., Ottosen C., Brikell I., Als T.D., Demontis D., Børglum A.D., Nordentoft M., Mors O., Werge T., Hougaard D., Bybjerg-Grauholm J., Hansen M.B., Mortensen P.B., Thapar A., Riglin L., Langley K., Dalsgaard S. (2020). Genetic liability to ADHD and substance use disorders in individuals with ADHD.. Addiction.

[53] Spencer A.E., Faraone S.V., Bogucki O.E., Pope A.L., Uchida M., Milad M.R., Spencer T.J., Woodworth K.Y., Biederman J. (2016). Examining the association between posttraumatic stress disorder and attention-deficit/hyperactivity disorder: A systematic review and meta-analysis.. J. Clin. Psychiatry.

[54] Luderer M., Reinhard I., Richter A., Kiefer F., Weber T. (2020). ADHD is associated with a higher risk for traumatic events, self-reported PTSD, and a higher severity of PTSD symptoms in alcohol-dependent patients.. Eur. Addict. Res..

[55] Anker E., Haavik J., Heir T. (2020). Alcohol and drug use disorders in adult attention-deficit/hyperactivity disorder: Prevalence and associations with attention-deficit/hyperactivity disorder symptom severity and emotional dysregulation.. World J. Psychiatry.

[56] Kozak K., Lucatch A.M., Lowe D.J.E., Balodis I.M., MacKillop J., George T.P. (2019). The neurobiology of impulsivity and substance use disorders: Implications for treatment.. Ann. N. Y. Acad. Sci..

[57] Adan A., Forero D.A., Navarro J.F. (2017). Personality traits related to binge drinking: A systematic review.. Front. Psychiatry.

[58] Weafer J., Fillmore M.T., Milich R. (2009). Increased sensitivity to the disinhibiting effects of alcohol in adults with ADHD.. Exp. Clin. Psychopharmacol..

[59] Ivanov I., Parvaz M.A., Velthorst E., Shaik R.B., Sandin S., Gan G., Spechler P., Albaugh M.D., Chaarani B., Mackey S., Banaschewski T., Bokde A.L.W., Bromberg U., Büchel C., Quinlan E.B., Desrivières S., Flor H., Grigis A., Gowland P., Heinz A., Ittermann B., Martinot J.L., Paillère Martinot M.L., Artiges E., Lemaitre H., Nees F., Orfanos D.P., Paus T., Poustka L., Hohmann S., Millenet S., Fröhner J.H., Smolka M.N., Walter H., Whelan R., Schumann G., Garavan H., Rapp M., Artiges E., Schneider S., Paus T., Barbot A., Barker G., Bokde A., Vetter N., Büchel C., Cattrell A., Constant P., Gowland P., Crombag H., Dalley J., Decideur B., Spranger T., Ripley T., Heym N., Flor H., Sommer W., Fuchs B., Gallinat J., Garavan H., Spanagel R., Kaviani M., Heinrichs B., Andreas Heinz,, Subramaniam N., Jia T., Ihlenfeld A., Ireland J., Ittermann B., Conrod P., Banaschewski T., Jones J., Klaassen A., Lalanne C., Lanzerath D., Lawrence C., Lemaitre H., Desrivieres S., Mallik C., Mann K., Mar A., Martinez-Medina L., Martinot J-L., Mennigen E., Mesquita de Carvahlo F., Schwartz Y., Bruehl R., Müller K., Nees F., Nymberg C., Lathrop M., Robbins T., Pausova Z., Pentilla J., Biondo F., Poline J-B., Poustka L., Millenet S., Smolka M., Fröhner J., Struve M., Williams S., Hübner T., Bromberg U., Aydin S., Rogers J., Romanowski A., Schmäl C., Schmidt D., Ripke S., Arroyo M., Schubert F., Pena-Oliver Y., Fauth-Bühler M., Mignon X., Whelan R., Speiser C., Fadai T., Stephens D., Ströhle A., Paillere M-L., Strache N., Theobald D., Jurk S., Vulser H., Miranda R., Yacubilin J., Frouin V., Genauck A., Parchetka C., Gemmeke I., Kruschwitz J., WeiB K., Walter H., Feng J., Papadopoulos D., Filippi I., Ing A., Ruggeri B., Xu B., Macare C., Chu C., Hanratty E., Burke Quinlan E., Robert G., Schumann G., Yu T., Ziesch V., Stedman A. (2021). A. Substance use initiation, particularly alcohol, in drug-naive adolescents: Possible predictors and consequences from a large cohort naturalistic study.. J. Am. Acad. Child Adolesc. Psychiatry.

[60] Volkow N.D., Wang G-J., Telang F., Fowler J.S., Logan J., Jayne M. (2077). Profound decreases in dopamine release in striatum in detoxified alcoholics: Possible orbitofrontal involvement.. J Neurosci.

[61] Luo Y., Weibman D., Halperin J.M., Li X. (2019). A review of heterogeneity in attention deficit/hyperactivity disorder (ADHD).. Front Hum Neurosci..

[62] Cerullo M.A., Strakowski S.M. (2007). The prevalence and significance of substance use disorders in bipolar type I and II disorder.. Subst. Abuse Treat. Prev. Policy.

[63] Scott J., Grunze H., Meyer T.D., Nendick J., Watkins H., Ferrier N. (2015). A bipolar II cohort (ABC): The association of functional disability with gender and rapid cycling.. J. Affect. Disord..

[64] Begleiter H., Reich T., Nurnberger J., Li T.K., Conneally P.M., Edenberg H., Crowe R., Kuperman S., Schuckit M., Bloom F., Hesselbrock V., Porjesz B., Cloninger C.R., Rice J., Goate A. (1999). Description of the genetic analysis workshop 11 collaborative study on the genetics of alcoholism.. Genet. Epidemiol..

[65] Hunt G.E., Malhi G.S., Cleary M., Lai H.M.X., Sitharthan T. (2016). Comorbidity of bipolar and substance use disorders in national surveys of general populations, 1990–2015: Systematic review and meta-analysis.. J. Affect. Disord..

[66] Frye M.A., Salloum I.M. (2006). Bipolar disorder and comorbid alcoholism: Prevalence rate and treatment considerations.. Bipolar Disord..

[67] Balanzá-Martínez V., Crespo-Facorro B., González-Pinto A., Vieta E. (2015). Bipolar disorder comorbid with alcohol use disorder: Focus on neurocognitive correlates.. Front. Physiol..

[68] Rakofsky J.J., Dunlop B.W. (2013). Do alcohol use disorders destabilize the course of bipolar disorder?. J. Affect. Disord..

[69] Manwani S.G., Szilagyi K.A., Zablotsky B., Hennen J., Griffin M.L., Weiss R.D. (2007). Adherence to pharmacotherapy in bipolar disorder patients with and without co-occurring substance use disorders.. J. Clin. Psychiatry.

[70] Baldessarini R.J., Perry R., Pike J. (2008). Factors associated with treatment nonadherence among US bipolar disorder patients.. Hum. Psychopharmacol..

[71] Lingam R., Scott J. (2002). Treatment non-adherence in affective disorders.. Acta Psychiatr. Scand..

[72] Stilley C.S., Bender C.M., Dunbar-Jacob J., Sereika S., Ryan C.M. (2010). The impact of cognitive function on medication management: Three studies.. Health Psychol..

[73] Agosti V., Chen Y., Levin F.R. (2011). Does attention deficit hyperactivity disorder increase the risk of suicide attempts?. J. Affect. Disord..

[74] Furczyk K., Thome J. (2014). Adult ADHD and suicide.. Atten. Defic. Hyperact. Disord..

[75] Sher L. (2006). Alcoholism and suicidal behavior: A clinical overview.. Acta Psychiatr. Scand..

[76] Hufford M.R. (2001). Alcohol and suicidal behavior.. Clin. Psychol. Rev..

[77] Pompili M., Gonda X., Serafini G., Innamorati M., Sher L., Amore M., Rihmer Z., Girardi P. (2013). Epidemiology of suicide in bipolar disorders: A systematic review of the literature.. Bipolar Disord..

[78] Miller J.N., Black D.W. (2020). Bipolar disorder and suicide: A review.. Curr. Psychiatry Rep..

[79] Goldberg J.F., Garno J.L., Leon A.C., Kocsis J.H., Portera L. (1999). A history of substance abuse complicates remission from acute mania in bipolar disorder.. J. Clin. Psychiatry.

[80] Geller B., Cooper T.B., Sun K., Zimerman B., Frazier J., Williams M., Heath J. (1998). Double-blind and placebo-controlled study of lithium for adolescent bipolar disorders with secondary substance dependency.. J. Am. Acad. Child Adolesc. Psychiatry.

[81] Preuss U.W., Schaefer M., Born C., Grunze H. (2021). Bipolar disorder and comorbid use of illicit substances.. Medicina (Kaunas).

[82] Angst J., Gamma A., Endrass J., Rössler W., Ajdacic-Gross V., Eich D., Herrell R., Merikangas K.R. (2006). Is the association of alcohol use disorders with major depressive disorder a consequence of undiagnosed bipolar-II disorder?. Eur. Arch. Psychiatry Clin. Neurosci..

[83] Albanese M.J., Clodfelter R.C., Pardo T.B., Ghaemi S.N. (2006). Underdiagnosis of bipolar disorder in men with substance use disorder.. J. Psychiatr. Pract..

[84] Kelly T. (2018). Prospective: Is bipolar disorder being overdiagnosed?. Int. J. Methods Psychiatr. Res..

[85] Akiskal H.S., Benazzi F. (2006). The DSM-IV and ICD-10 categories of recurrent [major] depressive and bipolar II disorders: Evidence that they lie on a dimensional spectrum.. J. Affect. Disord..

[86] Salloum I.M., Brown E.S. (2017). Management of comorbid bipolar disorder and substance use disorders.. Am. J. Drug Alcohol Abuse.

[87] Perugi G., Pallucchini A., Rizzato S., De Rossi P., Sani G., Maremmani A.G.I., Pinzone V., Maremmani I. (2019). Pharmacotherapeutic strategies for the treatment of attention-deficit hyperactivity (ADHD) disorder with comorbid substance-use disorder (SUD).. Expert Opin. Pharmacother..

[88] Groenman A.P., Schweren L.J.S., Weeda W., Luman M., Noordermeer S.D.S., Heslenfeld D.J., Franke B., Faraone S.V., Rommelse N., Hartman C.A., Hoekstra P.J., Buitelaar J., Oosterlaan J. (2019). Stimulant treatment profiles predicting co-occurring] substance use disorders in individuals with attention-deficit/] hyperactivity disorder.. Eur. Child Adolesc. Psychiatry.

[89] Groenman A.P., Oosterlaan J., Rommelse N.N.J., Franke B., Greven C.U., Hoekstra P.J., Hartman C.A., Luman M., Roeyers H., Oades R.D., Sergeant J.A., Buitelaar J.K., Faraone S.V. (2013). Stimulant treatment for attention-deficit hyperactivity disorder and risk of developing substance use disorder.. Br. J. Psychiatry.

[90] Wilens T.E., Adamson J., Monuteaux M.C., Faraone S.V., Schillinger M., Westerberg D., Biederman J. (2008). Effect of prior stimulant treatment for attention-deficit/hyperactivity disorder on subsequent risk for cigarette smoking and alcohol and drug use disorders in adolescents.. Arch. Pediatr. Adolesc. Med..

[91] Özgen H., Spijkerman R., Noack M., Holtmann M., Schellekens A., Dalsgaard S., van den Brink W., Hendriks V. (2021). Treatment of adolescents with concurrent substance use disorder and attention-deficit/hyperactivity disorder: A systematic review.. J. Clin. Med..

[92] Mannuzza S., Klein R.G., Truong N.L., Moulton J.L., Roizen E.R., Howell K.H., Castellanos F.X. (2008). Age of methylphenidate treatment initiation in children with ADHD and later substance abuse: Prospective follow-up into adulthood.. Am. J. Psychiatry.

[93] Salloum I.M., Cornelius J.R., Daley D.C., Kirisci L., Himmelhoch J.M., Thase M.E. (2005). Efficacy of valproate maintenance in patients with bipolar disorder and alcoholism: A double-blind placebo-controlled study.. Arch. Gen. Psychiatry.

[94] Kemp D.E., Gao K., Ganocy S.J., Elhaj O., Bilali S.R., Conroy C., Findling R.L., Calabrese J.R. (2009). A 6-month, double-blind, maintenance trial of lithium monotherapy versus the combination of lithium and divalproex for rapid-cycling bipolar disorder and Co-occurring substance abuse or dependence.. J. Clin. Psychiatry.

[95] Brady K.T., Myrick H., Henderson S., Coffey S.F. (2002). The use of divalproex in alcohol relapse prevention: A pilot study.. Drug Alcohol Depend..

[96] McMillan T.M. (1981). Lithium and the treatment of alcoholism: A critical review.. Addiction.

[97] Fawcett J., Clark D.C., Aagesen C.A., Pisani V.D., Tilkin J.M., Sellers D., McGuire M., Gibbons R.D. (1987). A double-blind, placebo-controlled trial of lithium carbonate therapy for alcoholism.. Arch. Gen. Psychiatry.

[98] Clark D.C., Fawcett J. (1989). Does lithium carbonate therapy for alcoholism deter relapse drinking?. Recent Dev. Alcohol..

[99] Gadh S. (2020). Low-dose lithium impact in an addiction treatment setting.. Pers. Med. Psychiatry.

[100] Rubio G., López-Muñoz F., Alamo C. (2006). Effects of lamotrigine in patients with bipolar disorder and alcohol dependence.. Bipolar Disord..

[101] Brown E.S., Nejtek V.A., Perantie D.C., Orsulak P.J., Bobadilla L. (2003). Lamotrigine in patients with bipolar disorder and cocaine dependence.. J. Clin. Psychiatry.

[102] Brown E.S., Perantie D.C., Dhanani N., Beard L., Orsulak P., Rush A.J. (2006). Lamotrigine for bipolar disorder and comorbid cocaine dependence: A replication and extension study.. J. Affect. Disord..

[103] Brady K.T., Sonne S.C., Malcolm R.J., Randall C.L., Simpson K., Dansky B.S., Roberts J.S., Brondino M. (2002). Carbamazepine in the treatment of cocaine dependence: Subtyping by affective disorder.. Exp. Clin. Psychopharmacol..

[104] Martinotti G., Di Nicola M., Romanelli R., Andreoli S., Pozzi G., Moroni N., Janiri L. (2007). High and low dosage oxcarbazepine versus naltrexone for the prevention of relapse in alcohol-dependent patients.. Hum. Psychopharmacol..

[105] Croissant B., Diehl A., Klein O., Zambrano S., Nakovics H., Heinz A., Mann K. (2006). A pilot study of oxcarbazepine versus acamprosate in alcohol-dependent patients.. Alcohol. Clin. Exp. Res..

[106] Croissant B., Scherle T., Diehl A., Heinz A., Mann K. (2004). Oxcarbazepine in alcohol relapse prevention-a case series..

[107] Martinotti G., Romanelli R., Di Nicola M., Reina D., Mazza M., Janiri L. (2007). Oxcarbazepine at high dosages for the treatment of alcohol dependence.. Am. J. Addict..

[108] Sylvia L.G., Gold A.K., Stange J.P., Peckham A.D., Deckersbach T., Calabrese J.R., Weiss R.D., Perlis R.H., Nierenberg A.A., Ostacher M.J. (2016). A randomized, placebo-controlled proof-of-concept trial of adjunctive topiramate for alcohol use disorders in bipolar disorder.. Am. J. Addict..

[109] Chiesa A., Chierzi F., De Ronchi D., Serretti A. (2012). Quetiapine for bipolar depression.. Int. Clin. Psychopharmacol..

[110] Brown E.S., Davila D., Nakamura A., Carmody T.J., Rush A.J., Lo A., Holmes T., Adinoff B., Caetano R., Swann A.C., Sunderajan P., Bret M.E. (2014). A randomized, double-blind, placebo-controlled trial of quetiapine in patients with bipolar disorder, mixed or depressed phase, and alcohol dependence.. Alcohol. Clin. Exp. Res..

[111] Stedman M., Pettinati H.M., Brown E.S., Kotz M., Calabrese J.R., Raines S. (2010). A double-blind, placebo-controlled study with quetiapine as adjunct therapy with lithium or divalproex in bipolar I patients with coexisting alcohol dependence.. Alcohol. Clin. Exp. Res..

[112] Brown E.S., Garza M., Carmody T.J. (2008). A randomized, double-blind, placebo-controlled add-on trial of quetiapine in outpatients with bipolar disorder and alcohol use disorders.. J. Clin. Psychiatry.

[113] Corbo M., Martinotti G., Aguglia A., Salvi V., Amerio A., Calò S., Fusar-Poli L., Serafini G., Signorelli M., Amore M., Mencacci C., Di Sciascio G., Biggio G., Aguglia E., Di Giannantonio M. (2021). Long‐acting second‐generation and oral antipsychotics for substance use disorders and psychotic symptoms: Prescribing attitudes among Italian psychiatrists.. Perspect. Psychiatr. Care.

[114] Brown E.S., Beard L., Dobbs L., Rush A.J. (2006). Naltrexone in patients with bipolar disorder and alcohol dependence.. Depress. Anxiety.

[115] Brown E.S., Carmody T.J., Schmitz J.M., Caetano R., Adinoff B., Swann A.C., Rush A.J. (2009). A randomized, double-blind, placebo-controlled pilot study of naltrexone in outpatients with bipolar disorder and alcohol dependence.. Alcohol. Clin. Exp. Res..

[116] Tolliver B.K., DeSantis S.M., Brown D.G., Prisciandaro J.J., Brady K.T. (2012). A randomized, double-blind, placebo-controlled clinical trial of acamprosate in alcohol-dependent individuals with bipolar disorder: A preliminary report.. Bipolar Disord..

[117] Quinn P.D., Chang Z., Hur K., Gibbons R.D., Lahey B.B., Rickert M.E., Sjölander A., Lichtenstein P., Larsson H., D’Onofrio B.M. (2017). ADHD Medication and Substance-Related Problems.. Am. J. Psychiatry.

[118] Wilens T.E., Adler L.A., Weiss M.D., Michelson D., Ramsey J.L., Moore R.J., Renard D., Brady K.T., Trzepacz P.T., Schuh L.M., Ahrbecker L.M., Levine L.R. (2008). Atomoxetine treatment of adults with ADHD and comorbid alcohol use disorders.. Drug Alcohol Depend..

[119] Manni C., Cipollone G., Pallucchini A., Maremmani A.G.I., Perugi G., Maremmani I. (2019). Remarkable reduction of cocaine use in dual disorder (Adult Attention Deficit Hyperactive Disorder/] Cocaine Use Disorder) patients treated with medications for ADHD.. Int. J. Environ. Res. Public Health.

[120] Konstenius M., Jayaram-Lindström N., Guterstam J., Beck O., Philips B., Franck J. (2014). Methylphenidate for attention deficit hyperactivity disorder and drug relapse in criminal offenders with substance dependence: A 24‐week randomized placebo‐controlled trial.. Addiction.

[121] Kast K.A., Rao V., Wilens T.E. (2021). Pharmacotherapy for attentiondeficit/ hyperactivity disorder and retention in outpatient substance use disorder treatment.. J. Clin. Psychiatry.

[122] Pallucchini A., Carli M., Maremmani A., Scarselli M., Perugi G., Maremmani I. (2021). Influence of substance use disorder on treatment retention of adult-attention-deficit/hyperactive disorder patients. A 5-Year follow-up study.. J. Clin. Med..

[123] Perugi G., Vannucchi G., Bedani F., Favaretto E. (2017). Use of stimulants in bipolar disorder.. Curr. Psychiatry Rep..

[124] Salvi V., Ribuoli E., Servasi M., Orsolini L., Volpe U. (2021). ADHD and bipolar disorder in adulthood: Clinical and treatment implications.. Medicina (Kaunas).

[125] Kumar V., Varambally S. (2017). Atomoxetine induced hypomania in a patient with bipolar disorder and adult attention deficit hyperactivity disorder.. Indian J. Psychol. Med..

[126] Wang L.J., Shyu Y.C., Yuan S.S., Yang C.J., Yang K.C., Lee T.L., Lee S.Y. (2016). Attention-deficit hyperactivity disorder, its pharmacotherapy, and the risk of developing bipolar disorder: A nationwide population-based study in Taiwan.. J. Psychiatr. Res..

[127] Lydon E., El-Mallakh R.S. (2006). Naturalistic long-term use of methylphenidate in bipolar disorder.. J. Clin. Psychopharmacol..

[128] Carlson P.J., Merlock M.C., Suppes T. (2004). Adjunctive stimulant use in patients with bipolar disorder: Treatment of residual depression and sedation.. Bipolar Disord..

[129] Viktorin A., Rydén E., Thase M.E., Chang Z., Lundholm C., D’Onofrio B.M., Almqvist C., Magnusson P.K.E., Lichtenstein P., Larsson H., Landén M. (2017). The risk of treatment-emergent mania with methylphenidate in bipolar disorder.. Am. J. Psychiatry.

[130] Yatham L.N., Kennedy S.H., Parikh S.V., Schaffer A., Bond D.J., Frey B.N., Sharma V., Goldstein B.I., Rej S., Beaulieu S., Alda M., MacQueen G., Milev R.V., Ravindran A., O’Donovan C., McIntosh D., Lam R.W., Vazquez G., Kapczinski F., McIntyre R.S., Kozicky J., Kanba S., Lafer B., Suppes T., Calabrese J.R., Vieta E., Malhi G., Post R.M., Berk M. (2018). Canadian Network for Mood and Anxiety Treatments (CANMAT) and International Society for Bipolar Disorders (ISBD) 2018 guidelines for the management of patients with bipolar disorder.. Bipolar Disord..

